# A Comprehensive Analysis Identified the Key Differentially Expressed Circular Ribonucleic Acids and Methylation-Related Function in Pheochromocytomas and Paragangliomas

**DOI:** 10.3389/fgene.2020.00015

**Published:** 2020-02-25

**Authors:** Anze Yu, Minghao Li, Changsheng Xing, Danlei Chen, Cikui Wang, Qiao Xiao, Liang Zhang, Yingxian Pang, Yong Wang, Xiongbing Zu, Longfei Liu

**Affiliations:** ^1^ Department of Urology, Xiangya Hospital, Central South University, Changsha, China; ^2^ National Clinical Research Center for Geriatric Disorders, Xiangya Hospital, Central South University, Changsha, China; ^3^ Center for Inflammation and Epigenetics, Houston Methodist Research Institute, Houston, TX, United States

**Keywords:** pheochromocytoma, paraganglioma, circular ribonucleic acid, histone methylation, epigenetics, biomarker

## Abstract

We investigated differentially expressed circular RNAs (circRNAs) and their potential functions in pheochromocytomas and paragangliomas (PCC/PGLs). Expression levels of circRNAs in tumor and adjacent normal tissues from seven PCC/PGL patients were analyzed through RNA sequencing. Real-time PCR was conducted to verify the key candidates identified in the sequencing data. Gene ontology (GO) and Kyoto Encyclopedia of Genes and Genomes (KEGG) pathway analyses were performed to predict the functions of these circRNAs. A total of 367 circRNAs were found differentially expressed between tumor and normal samples. The top three histone methylation-related circRNAs (hsa_circ_0000567, hsa_circ_0002897, and hsa_circ_0004473) and their target microRNAs (miRNAs) were identified and validated. We then mapped the circRNA-miRNA-messenger RNA (mRNA) coding-noncoding gene co-expression (CNC) networks to show the potential binding relationships between circRNAs and their targets in PCC/PGLs. The top five mRNAs, 88 miRNAs, and 132 circRNAs related to pathogenesis were utilized to map the CNC network, and we observed that the interactions of these candidates with their target miRNAs regulated histone methylation and further mediated PCC/PGL pathogenesis. This study is the first to provide the whole profile of differentially expressed circRNAs in PCC/PGLs. Our data indicate that altered circRNAs may control the pathogenesis of PCC/PGLs by regulating histone methylation processes, highlighting their role as potential biomarkers.

## Introduction

Pheochromocytomas and paragangliomas (PCC/PGLs) are highly genetically related, neuroendocrine tumors that have been listed as rare diseases by the World Health Organization (Neumann et al., 2019). Adrenal chromaffin cells secrete catecholamines and give rise to PCC/PGLs (Neumann et al., 2007; Neary et al., 2011; Gill et al., 2011). Approximately 90% of chromaffin cells are present in the adrenal medulla and tumors that occur here are termed PCC (Ferreira et al., 2016). The other 10% of cells are located outside the adrenal medulla, such as in the heart, bladder, and neck, and these tumor cases are called extra-adrenal PCC or PGL (Crona et al., 2017). The major clinical manifestations of PCC/PGLs are unpredictable hypertension, cardiovascular crisis, hyperhidrosis, palpitations, and elevated basal metabolic rate, which are induced by the excessive secretion of catecholamines (Jochmanova and Pacak, 2016; Loper et al., 2016). PCC/PGLs are also involved in some severe syndromes such as von Hippel-Lindau disease (Liu P. et al., 2019), multiple endocrine neoplasia (type I and II), and neurofibromatosis (Rednam et al., 2017). If patients fail to undergo proper treatment in time, their 5-year survival rate drops below 40% due to excessive catecholamine secretion as well as the development of chemotherapy and radiation therapy resistance (Toledo et al., 2016; Taieb and Pacak, 2017; Li et al., 2018). As PCC/PGLs are among the most genetically related human cancers with 60% of patients showing family aggregation (Fishbein et al., 2017; Liu et al., 2018; Job et al., 2019), genetic screening is of great value and significance for the diagnosis and prevention of PCC/PGLs (Calsina et al., 2019). Moreover, over 15 genomic and transcriptomic molecules reported to regulate PCC/PGL development, such as *SDHx, VHL, TMEM127, HRAS, FGFR1, ATRX, RET, EPAS1, MAX, EGLN1*, and *NF1*, were found mutated in the germline of PCC/PGLs patients (Gill et al., 2011; Castro-Vega et al., 2015; Rednam et al., 2017; Taieb and Pacak, 2017; Remacha et al., 2017; Pang et al., 2018).

Circular RNAs (circRNAs) are highly evolutionarily conserved, regulatory, noncoding RNAs that play significant roles in various biological and pathological processes, such as gene translation, transcription, and tumor genesis and development; they also act as microRNA (miRNA) sponges. CircRNAs have a stable loop structure that lacks a 5’ cap and 3’ tail and are thus not easily degraded by RNase. Under specific circumstances, some circRNAs can open their loop structure for translation (Memczak et al., 2013; Liu C.X. et al., 2019). Moreover, circRNAs exhibit abnormal expression in numerous cancer types, including liver, stomach, breast, ovarian, and oral cancers; therefore, they have been considered novel biomarkers for tumor screening and are potential therapeutic targets (Hansen et al., 2013; Conn et al., 2015; Meng et al., 2019; Vo et al., 2019).

Considering the high genetic relevance and neuroendocrine characteristics of PCC/PGLs, we speculated that circRNAs may play important roles in regulating the development of PCC/PGLs. Previous studies have identified multiple key mediators for the pathogenesis of PCC/PGLs (Rednam et al., 2017; Remacha et al., 2017; Job et al., 2019), such as genetic alterations of *SHDx* and *VHL*, but whether these components are modulated by circRNAs remains unknown. Therefore, we performed RNA sequencing using tumor tissues and para-cancerous normal adrenal medulla tissues from PCC/PGLs patients to examine the aberrant expression of circRNAs and miRNAs. The data were evaluated by comparative analyses of reference sequences and tissue specificity was predicted. circRNAs with significantly altered expression were further screened and their binding sites to miRNAs predicted. In addition, we performed gene ontology (GO) and Kyoto Encyclopedia of Genes and Genomes (KEGG) pathway analyses using these differentially expressed circRNAs and mapped their interaction networks.

## Materials and Methods

### Sample Collection

Tumor and adjacent normal adrenal medulla specimens were collected during surgery from 40 PCC/PGL patients who underwent laparoscopic resection between February 2018 and September 2018 in Xianya Hospital. Detailed patient information is listed in **Table 1**. Seven PCC/PGL patients were randomly selected for RNA sequencing of their tissues while the other 33 PCC/PGL patients were used to validate the sequencing outcomes. All samples were validated by pathological examination and were frozen and stored immediately in liquid nitrogen until further use. Peripheral blood of 16 PCC/PGL patients and 16 healthy donors was collected and stored immediately at −80°C. All patients were primarily diagnosed with PCC/PGLs *via* imaging (computerized tomography and color ultrasound) and laboratory examination (urine vanillylmandelic acid), and did not undergo any treatments before surgery. All patients signed informed consent forms before the surgery and study, which was approved by the Ethics Committee of Xiangya Hospital of Central South University.

**Table 1:** Characteristics of the seven pheochromocytomas and paragangliomas (PCC/PGL) patients whose samples were used for RNA sequencing.

e	Sex	Age	Blood pressure before surgery	VMA (U)	Pathological diagnosis	Immunohistochemistry	Genotype (gene mutation)
1	Female	53	230/100	80.1	PCC (right adrenal gland)	CgA (+), Syn (+), S-100 (−), SF-1 (−), Ki67 (1%+), inhibin (−), Melan-A (−), P53 (−)	HRAS
2	Female	52	114/79	34.8	PCC (right adrenal gland)	CgA (+), Syn (+), S-100 (−), SF-1 (−), Ki67 (1%+), inhibin (−), Melan-A (−), P53 (−)	RET
3	Male	32	140/87	Normal	PCC (left adrenal gland)	CgA (+), Syn (++), S-100 (+), Ki67 (2%+), inhibin (+), Melan-A (-), P53 (−)	VHL
4	Male	8	210/130	97/21.6	PCC (double adrenal glands)	CgA (++), Syn (++), S-100 (++), Ki67 (2%+), inhibin (+), Melan-A (−), P53 (−)	RET/VHL
5	Male	49	129/88	89.9	PCC (right adrenal gland)	CgA (++), Syn (++), S-100 (++), Ki67 (2%+), inhibin (+), Melan-A (−), P53 (−)	VHL
6	Male	57	123/72	40/16.8	PCC (right adrenal gland)	CgA (++), Syn (+), S-100 (+), SF-1 (−), Ki67 (< 1%+), inhibin (−), Melan-A (−), P53 (−)	HRAS
7	Female	51	141/83	58.2	PCC (right adrenal gland)	CgA (++), Syn (+), S-100 (+), SF-1 (−), Ki67 (2%+), inhibin (−), Melan-A (−), P53 (−)	VHL

VMA, urine vanillylmandelic acid.

### Total Ribonucleic Acid Extraction

Frozen samples and peripheral blood were taken out of liquid nitrogen storage and immediately mixed with TRIzol reagent (Ambion, Austin, TX). Tissues were homogenized and lysed in TRIzol reagent, after which total RNAs were extracted according to manufacturer’s instructions.

### Ribonucleic Acid Quantification and Qualification

Electrophoresis on 2% agarose gels was used to determine whether the total RNAs were degraded or contaminated. A NanoPhotometer^®^ spectrophotometer (IMPLEN, Westlake Village, CA) and Qubit^®^ RNA Assay Kit in Qubit^®^2.0 Fluorometer (Life Technologies, Carlsbad, CA) were used to detect the purity and concentration of RNA, respectively. The RNA Nano6000 Assay Kit (Agilent Technologies, Santa Clara, CA) was used to assess RNA integrity.

### Ribonucleic Acid Library Construction, Clustering, and Sequencing

We used approximately 5 ug of total RNA for each sample and used the Ribo-Zero™ rRNA Removal Kit (Illumina, San Diego, CA) to eliminate ribosomal RNA interference according to manufacturer’s instructions. The residue was purified by two rounds of ethanol precipitation, after which divalent cations were used to splice the remaining RNAs into small fragments under high temperature. The small RNA fragments were reverse-transcribed to complementary DNA (cDNA) and second strand cDNA was synthesized using *Escherichia coli* DNA polymerase I, deoxyuridine triphosphates (dUTPs), and RNase H. After adding an A-base to the 3ʹ ends of each cDNA fragment and ligating to the NEBNext Adaptor [New England Biolabs (NEB), Ipswich, MA], we purified the library using the AMPureXP system (Beckman Coulter, Brea, CA) and prepared for hybridization. Next, approximately 2 µl USER enzyme (NEB) was added to the cDNA buffer for PCR. TruSeq SR Cluster Kit v3-cBot-HS (Illumia) was used to cluster the samples on the cBot Cluster Generation System. Finally, the Illumina HiSeq 2500/2000 sequencing platform was used to sequence the libraries (150 bp paired-end reads) after cluster generation. Sequencing data were analyzed against the reference genome, after which the circRNAs were filtered out. The software Find_circ and CIRI2 (Zeng et al., 2017) were used to identify circRNAs;

### Identification of Differentially Expressed Circular Ribonucleic Acids, Microribonucleic Acids, and Messenger Ribonucleic Acids

DESeq R package(1.8.3) (https://www.bioconductor.org) was used to perform read-count analysis of differentially expressed RNAs [circRNAs, miRNAs, and messenger RNAs (mRNAs)]. An adjusted p value of < 0.05 was considered statistically significant and related RNAs were recognized as differentially expressed. miRanda software (https://omictools.com › miranda-tool) was used to predict the target miRNA binding sites of circRNAs.

### Gene Ontology and Kyoto Encyclopedia of Genes and Genomes Pathway Enrichment Analysis

Gene Ontology (GO) enrichment analysis (http://www.webgestalt.org), an online software, was used to predict the molecular function, biological process, cellular components, and location of the target miRNAs of differentially expressed circRNAs. Kyoto Encyclopedia of Genes and Genomes (KEGG) pathway analysis (http://kobas.cbi.pku.edu.cn/anno_iden.php) was used to predict the possible metabolic pathways of some differentially expressed circRNAs Enrichment with p < 0.05 was regarded as statistically significant.

### Real Time Polymerase Chain Reaction Analysis

Reverse transcription of total RNA was performed using the Superscript IV Reverse Transcriptase (Thermo Fisher Scientific, Waltham, MA) at 50°C for 15 min to obtain cDNA. Real-time PCR was then performed using SYBR Green Reagent on an ABI PRISM 7500 Fast Real-Time PCR System (Thermo Fisher Scientific, Waltham, MA). Primers used for qPCR were synthesized at Sango Biotech (Shanghai, China) and are listed in **Supplementary Table 6**. Relative expression of the different genes was normalized to β-actin expression, and arbitrary units were used to display normalized gene expression. The data were analyzed using the 2^−ΔΔCt^ method.

### Circular Ribonucleic Acid-Microribonucleic Acid-Messenger Ribonucleic Acid Coding-Noncoding Gene Co-Expression Network Analysis

Cytoscape software (Shannon et al., 2003) was used to construct the circRNA-miRNA-mRNA networks.

### Statistical Analysis

All data were analyzed using SPSS version 23 (IBM, Armonk, NY) and GraphPad Prism 5 (GraphPad Software Inc., La Jolla, CA). A paired Student’s *t*-test was used to test significance of the results and p values < 0.05 were considered statistically significant.

## Results

### Circular Ribonucleic Acid Expression Profiles of Human Pheochromocytomas and Paragangliomas Tumor Tissues

**Figure 1:**
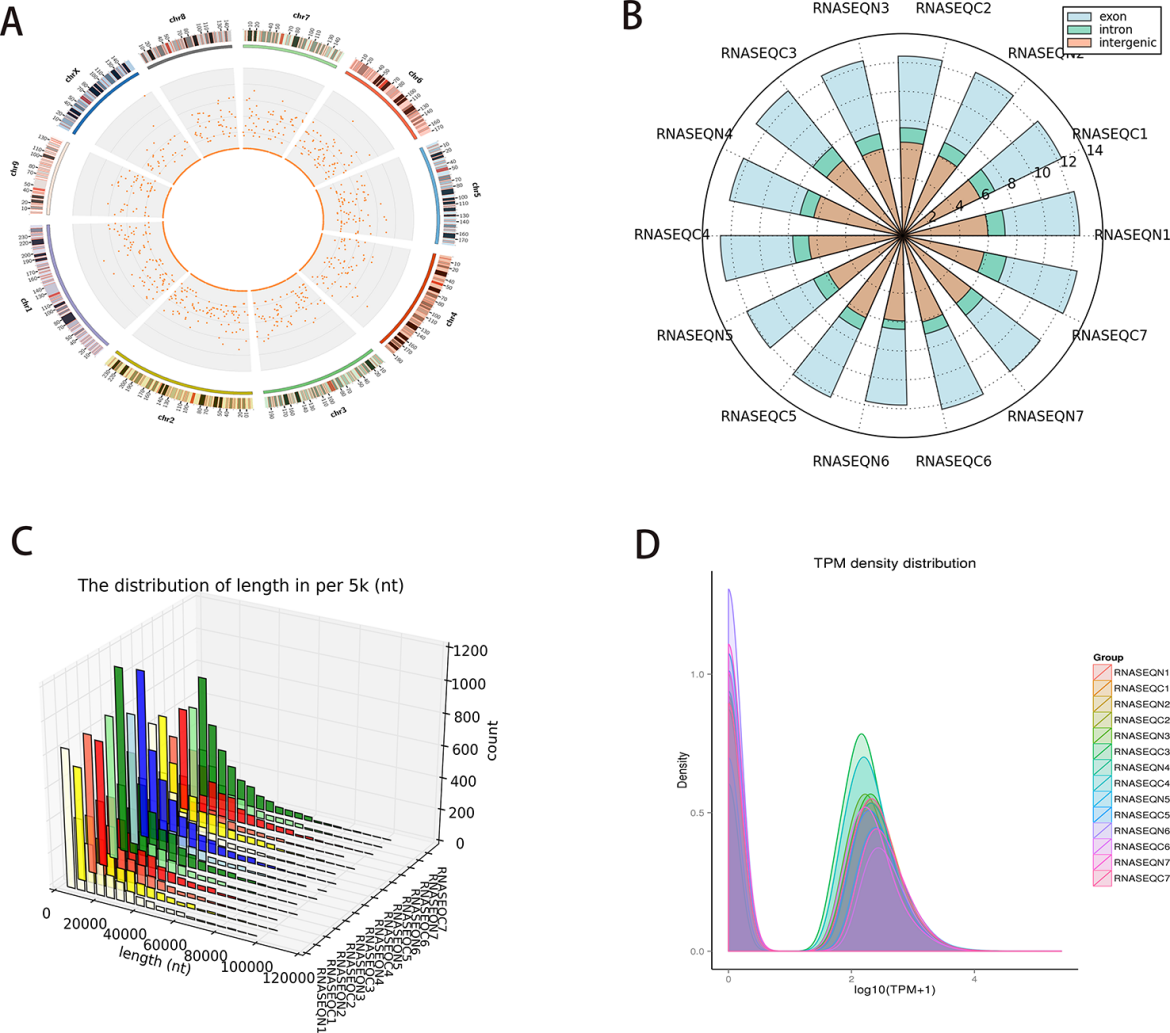
Profiles of circular RNAs (circRNAs) identified in pheochromocytomas and paragangliomas (PCC/PGLs). **(A)** Density distribution of circRNAs on the chromosome. **(B)** The different sources of circRNAs (exons, introns, and intergenic regions). **(C)** Length distribution graph of circRNAs in different patient samples. **(D)** Transcription per million (TPM) distribution graph. (RNASEQC: tumor samples of PCC/PGLs patients for RNA sequencing, RNASEQN: normal adrenal medulla tissues of PCC/PGLs patients for RNA sequencing).

The density distribution of circRNAs on the chromosome is shown in **Figure 1A**. (Chromosome 1–9 and X were shown in the panel, details of circRNAs on all the chromosome can be seen in **Supplementary Table 1** the density distribution of circRNAs on all the chromosome). Different sources (exons, introns, and intergenic regions) of circRNAs were counted (**Figure 1B**) because circRNAs can be acquired from the splicing of exons and introns. We thus speculated that most circRNAs in PCC/PGLs are obtained from the splicing of exons in both tumor and adjacent normal tissues. Next, we analyzed the length distribution of circRNAs in different patient samples (**Figure 1C**) and found that the majority of circRNAs were between 0 and 2,000 nt. Additionally, we mapped the transcription per million (TPM) distribution graph for an overall examination of the gene expression pattern in different samples (**Figure 1D**).

### Identification and Validation of Differentially Expressed Circular Ribonucleic Acid in Pheochromocytomas and Paragangliomas

**Figure 2:**
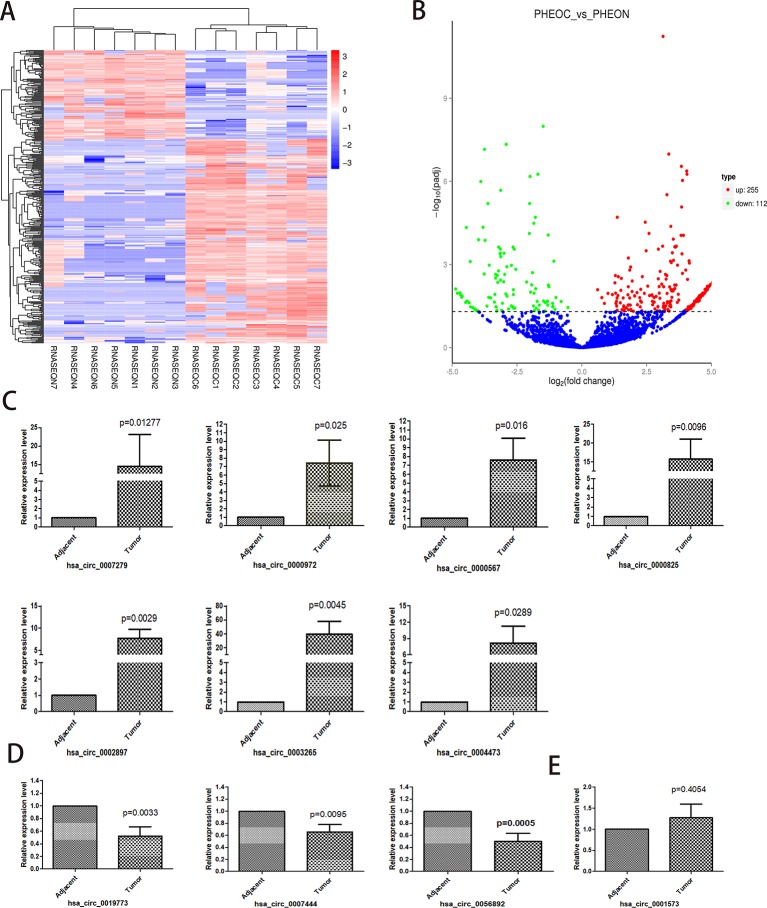
Identification and validation of differentially expressed circular RNAs (circRNAs) in pheochromocytomas and paragangliomas (PCC/PGLs). **(A)** Heatmap of differentially expressed circRNAs. (RNASEQC, tumor samples of PCC/PGLs patients for RNA sequencing; RNASEQN, normal adrenal medulla tissues of PCC/PGLs patients for RNA sequencing). **(B)** Volcano map of differentially expressed circRNAs; 255 were upregulated and 122 were downregulated. (PHEOC, tumor tissues of PCC/PGLs patients; PHEON, normal adrenal medulla tissues of PCC/PGLs patients.) **(C–E)** Real time PCR validation of the top 11 differentially expressed circRNAs in 33 pairs of PCC/PGL tumor tissues and adjacent tissues.

A total of 367 differentially expressed circRNAs were identified after RNA sequencing and qualification, among which 255 were upregulated and 112 were downregulated. The top 20 differentially expressed circRNAs are listed in **Table 2**. We next performed hierarchical cluster analysis of differentially expressed circRNAs and acquired the volcano map (log2FoldChange > 1; adjusted p < 0.05) and heat map for these samples (**Figures 2A, B**). We selected the top 11 differentially expressed circRNAs and conducted real-time qPCR using the other 33 pairs of PCC/PGLs samples to test if these circRNAs show the same relative expression as in the RNA sequencing data; information and genotype of the 33 patients is available in **Supplementary Table 2** information and genotype of the 33 patients and the primer sequences for each circRNA is listed in **Supplementary Table 6**. As shown in **Figure 2C–E**, expression levels of hsa_circ_0000567, hsa_circ_0002897, hsa_circ_0004473, hsa_circ_0000972, hsa_circ_0000825, hsa_circ_0003265, and hsa_circ_0007279 were significantly higher in tumor tissues than in para-cancerous tissues, whereas the expression of hsa_circ_0056892, hsa_circ_0019773, and hsa_circ_0007444 was markedly downregulated in tumors tissues compared with adjacent tissues. However, there was no difference in hsa_circ_0001573 expression levels *via* real-time PCR. Overall, these data indicate that the sequencing outcomes were reliable and that these altered circRNAs may play important roles in tumor genesis or pathological processes and can serve as potential biomarkers or therapeutic targets.

**Table 2:** The top 20 differentially expressed circular ribonucleic acids (circRNAs) in pheochromocytomas and paragangliomas.

ID	PHEOC_readcount	PHEON_readcount	log_2_FoldChange	p-val	p-adj
hsa_circ_0003265	74.30462	8.195594	3.1329	1.77E−15	5.90E−12
novel_circ_0010710	0.379333	48.06986	−6.1755	1.78E−14	2.96E−11
hsa_circ_0007444	72.18526	204.4537	−1.4916	9.31E−12	1.03E−08
hsa_circ_0019773	3.831636	32.74755	−2.9191	5.55E−11	4.62E−08
hsa_circ_0056892	1.305188	22.93686	−3.7578	1.05E−10	7.02E−08
novel_circ_0018500	65.11106	5.843535	3.3556	1.87E−10	1.04E−07
hsa_circ_0000825	20.28743	0.373391	5.4407	3.32E−10	1.58E−07
hsa_circ_0000972	40.7458	2.594112	3.8421	6.92E−10	2.88E−07
hsa_circ_0007279	23.44449	1.258304	4.047	1.15E−09	4.25E−07
hsa_circ_0001573	13.74913	46.72078	−1.6969	1.83E−09	5.53E−07
novel_circ_0012887	46.11258	2.453379	4.0571	1.68E−09	5.53E−07
novel_circ_0013677	10.2773	42.46868	−1.9987	2.41E−09	6.68E−07
novel_circ_0013545	29.82766	1.865443	3.8841	3.63E−09	9.30E−07
hsa_circ_0072391	2.601763	47.52957	−3.8994	4.27E−09	1.02E−06
novel_circ_0000068	2.383841	23.84816	−3.1286	9.48E−09	2.10E−06
novel_circ_0004082	24.64715	0.373391	5.4857	1.10E−08	2.30E−06
hsa_circ_0001633	132.6587	12.47332	3.2827	1.55E−08	3.05E−06
hsa_circ_0015454	0.891954	14.12767	−3.6241	3.43E−08	6.33E−06
hsa_circ_0077736	7.951543	33.60178	−2.0141	3.61E−08	6.33E−06
hsa_circ_0001678	27.54639	1.488962	3.8505	5.07E−08	8.44E−06

PHEOC, tumor tissues of PCC/PGLs patients; PHEON, normal adrenal medulla tissues of PCC/PGLs patients; p-adj, adjust p value, which is designed to control the expected proportion of “discoveries” (rejected null hypotheses) that are false (incorrect rejections).

### Gene Ontology Enrichment and Analysis of Genes Associated With Differentially Expressed Circular Ribonucleic Acids

**Figure 3:**
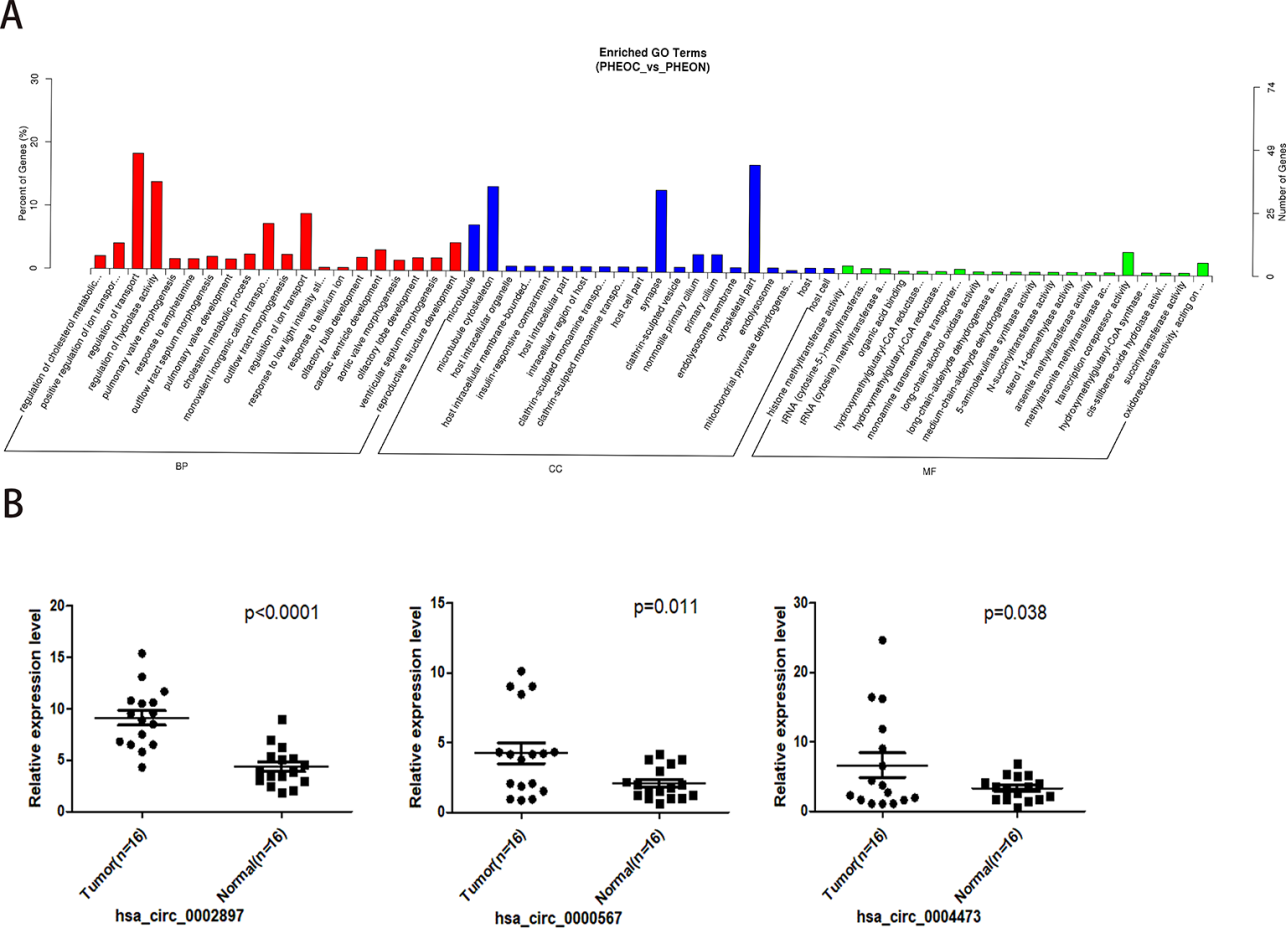
Gene ontology prediction and validation of histone methylation-related circular RNAs (circRNAs) in peripheral blood. **(A)** Gene ontology analysis of the top enriched molecular function of the differently expressed circRNAs. **(B)** Validation of the top three differentially expressed circRNAs by real time PCR using the peripheral blood of PCC/PGL patients and healthy donors.

After obtaining the differentially expressed circRNAs, we performed GO analysis on the source genes in each group based on the correspondence between circRNAs and their binding miRNAs and source genes (**Figure 3A**). We found that the main biological process terms were related to cell metabolism and transport, such as regulation of cholesterol metabolic process (GO:0090181), positive regulation of ion transport (GO:0043270), regulation of transport (GO:0051049), and regulation of hydrolase activity (GO:0051336). The highest enriched cellular component terms were associated with microtubule (GO:0005874) and microtubule cytoskeleton (GO:0015630). The most enriched molecular function terms were related to histone methyltransferase activity (H3-K36 specific; GO:0046975), transfer RNA (tRNA) (cytosine-5-)-methyltransferase activity (GO:0016428), and tRNA (cytosine) methyltransferase activity (GO:0016427).

GO enrichment results showed that the degree of enrichment of histone methylation-related circRNAs occupied the top positions of molecular function, suggesting that methylation regulation of target genes by circRNAs may play important roles in the pathogenesis of PCC/PGLs. We further performed real-time PCR to analyze the top three histone methylation-related circRNAs in the peripheral blood of 16 PCC/PGL patients (see **Supplementary Table 3** information and genotype of the 16 patients) and 16 healthy donors, and found that circRNAs has_circ_0000567, hsa_circ_0002897, ahashsa_circ_0004473 also exhibited relatively high expression levels in PCC/PGL patients compared with healthy donors (**Figure 3B**).

### Prediction and Analysis of the Histone Methylation-Related Circular Ribonucleic Acid-Microribonucleic Acid-Messenger Ribonucleic Acid Coding-Noncoding Gene Co-Expression Network

**Figure 4:**
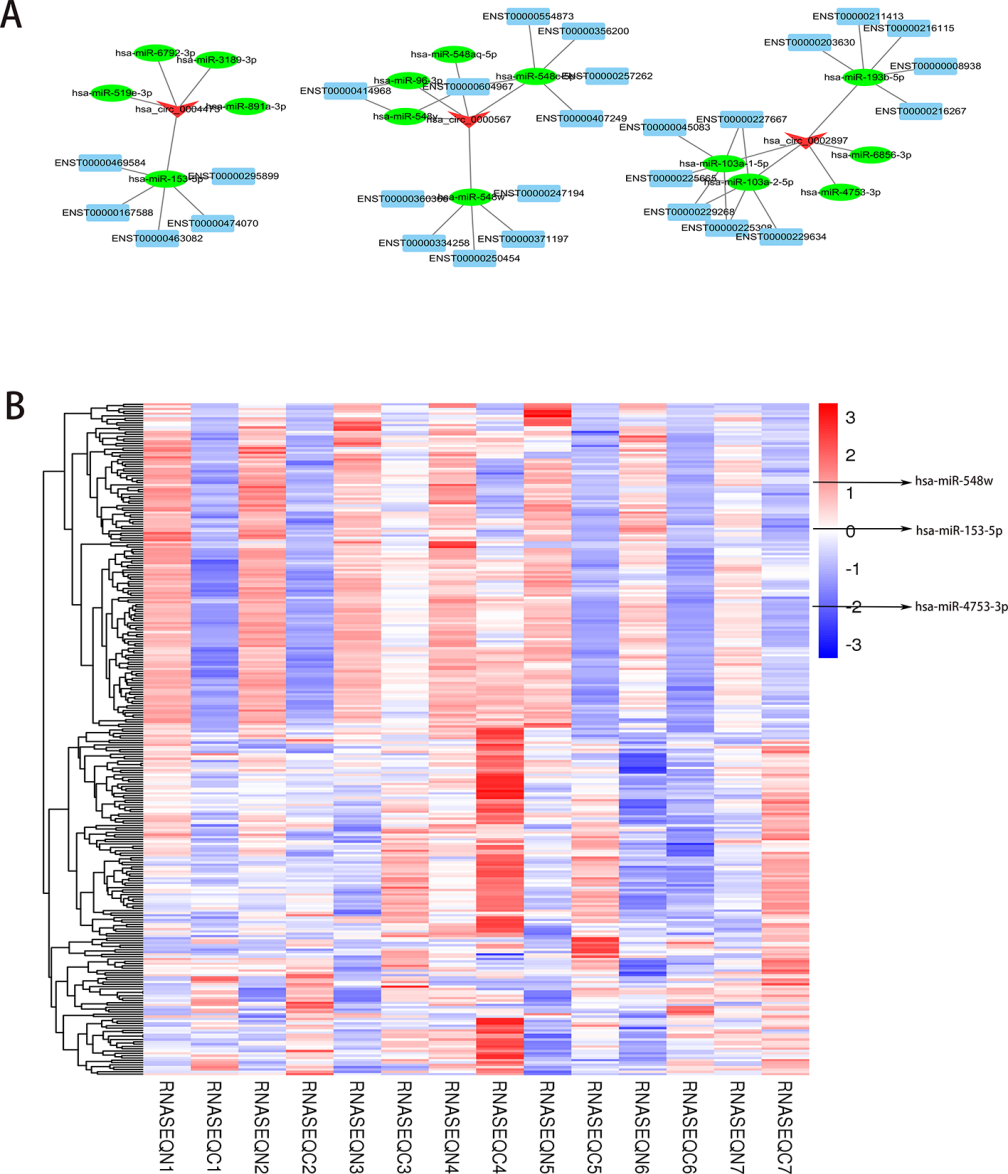
Prediction of histone methylation-related messenger RNAs (miRNAs). **(A)** The coding-noncoding gene co-expression (CNC) prediction network of the top three methylation-related circRNAs (hsa_circ_0000567, hsa_circ_0002897, and hsa_circ_0004473) and their target miRNAs and mRNAs (top five miRNAs and mRNAs are shown on the map). **(B)** Heatmap of differentially expressed miRNAs by RNA sequencing. (RNASEQC, tumor samples of PCC/PGLs patients for RNA sequencing; RNASEQN, normal adrenal medulla tissues of PCC/PGLs patients for RNA sequencing).

Next, we selected the three histone methylation-related circRNAs (hsa_circ_0000567, hsa_circ_0002897, and hsa_circ_0004473) with the most significant differences to predict and analyze their target miRNAs and mRNAs. The top five target miRNAs and related mRNAs were used to map the coding-noncoding gene co-expression (CNC) network (**Figure 4A**). Notably, the predicted binding miRNAs of the three upregulated circRNAs in PCC/PGL tumor tissues were all downregulated according to the miRNA sequencing data (**Figure 4B** and **Supplementary Table 4** the circRNAs-miRNAs binding relationship). Our findings suggest that these histone methylation-related circRNAs act as RNA sponges and regulate their target miRNAs, highlighting their potential as novel therapeutic targets for PCC/PGLs.

### Kyoto Encyclopedia of Genes and Genomes Pathway Enrichment and Analysis of Differentially Expressed Circular Ribonucleic Acids

**Figure 5:**
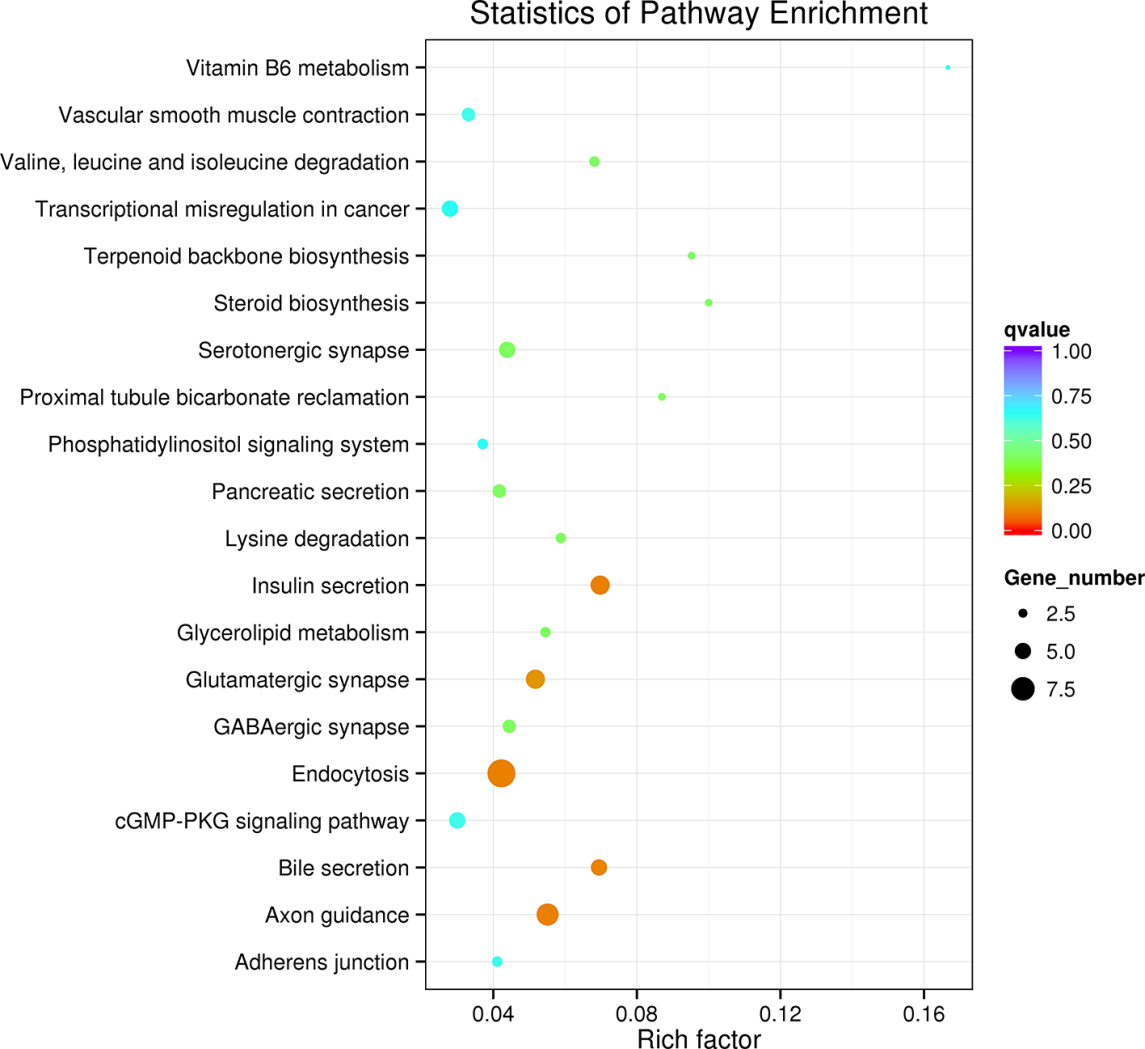
Kyoto Encyclopedia of Genes and Genomes (KEGG) analysis of the differently expressed circular RNAs (circRNAs) and their target gene-related enriched pathways.

KEGG pathway analysis revealed that the most enriched pathways were related to insulin secretion, axon guidance, endocytosis, bile secretion, glutamatergic synapse, serotonergic synapse, lysine degradation, and steroid biosynthesis. The top 20 enriched pathways were selected and displayed in a KEGG enrichment scatter plot diagram (**Figure 5**).

### Prediction and Analysis of the Relationship Between Known Pheochromocytoma and Paraganglioma Susceptibility Genes and the Circular Ribonucleic Acid-Microribonucleic Acid-Messenger Ribonucleic Acid Network

**Figure 6:**
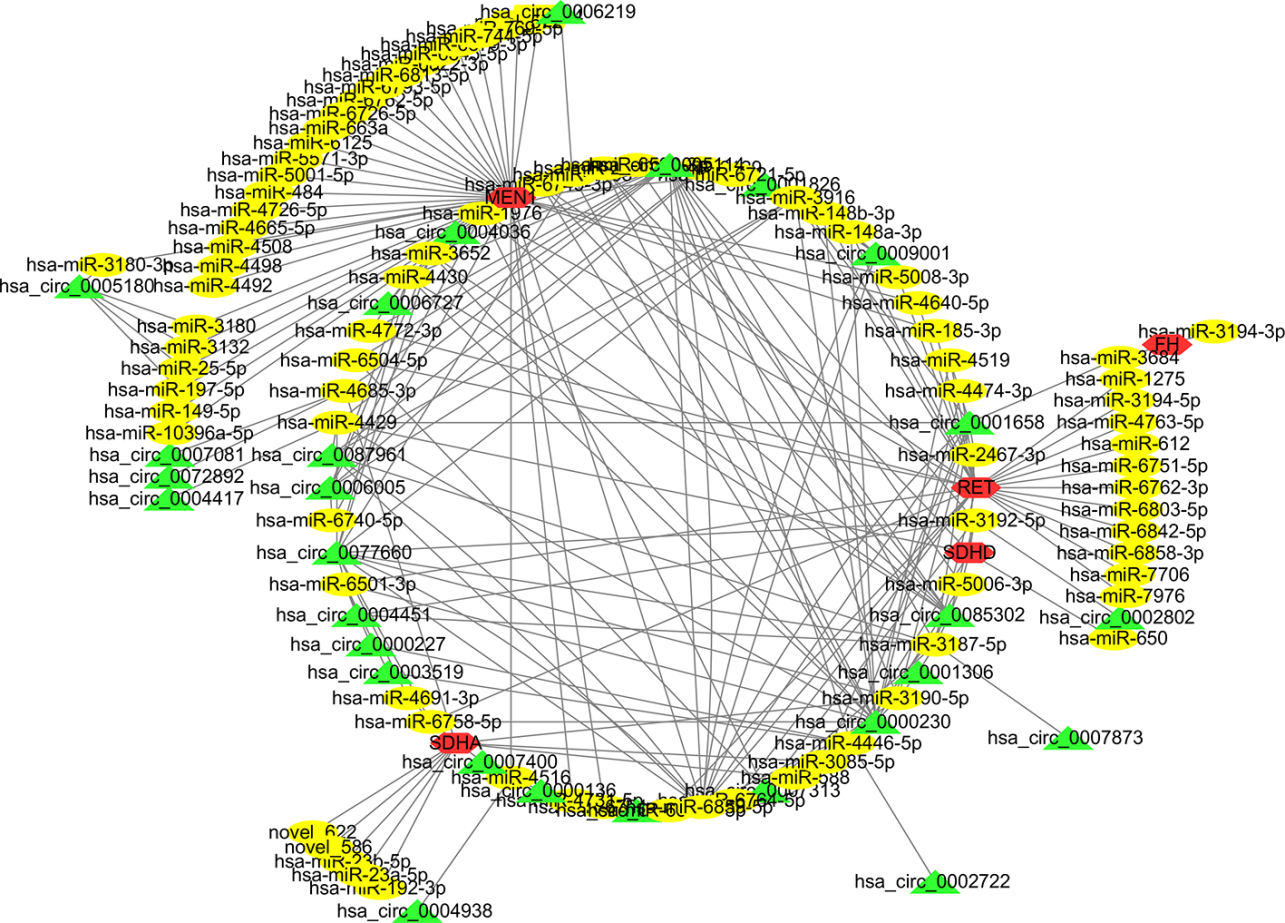
The competing endogenous RNA network of the known pheochromocytoma and paraganglioma (PCC/PGL) susceptibility genes associated with the circular ribonucleic acid-microribonucleic acid-messenger ribonucleic acid (circRNA-miRNA-mRNA) relationship. Five mRNAs (red), 88 miRNAs (yellow), and 132 circRNAs (green) were included in the analysis.

The currently known PCC/PGL-related susceptibility genes include *SDHx*, *VHL*, *TMEM127*, *HRAS*, *FGFR1*, *ATRX*, *RET*, *EPAS1*, *MAX*, *EGLN1*, and *NF1* (Castro-Vega et al., 2015). We thus examined whether the circRNAs exhibit regulatory relationships with the mRNA transcripts of these susceptibility genes. After analyzing and screening the mRNA sequencing data (**Supplementary Table 5** mRNA sequencing data of the 7 patients), we selected five differently expressed mRNAs, 88 miRNAs, and 132 circRNAs to map the CNC network. These findings support the role of circRNAs as RNA sponges and in regulating the expression levels of PCC/PGL susceptibility genes (**Figure 6**).

## Discussion

CircRNA is a special type of highly evolutionarily conserved, regulatory, noncoding RNA, which was originally considered noise or a redundancy of the transcription process (Memczak et al., 2013). However, an increasing number of studies demonstrated that circRNAs play important roles in various pathological or physiological processes (Zeng et al., 2017; Meng et al., 2019; Kolling et al., 2019; Kristensen et al., 2019; Li et al., 2019; Liang et al., 2019). Studies showed that circRNAs are mainly produced by RNA cleavage, are widely present throughout eukaryotic cells, and have high stability, species conservation, and tissue specificity. CircRNAs are also involved in intracellular RNA regulation networks and are closely associated with the development of many diseases, including systemic sclerosis and several cancer types, such as liver, lung, stomach, oral, nasopharyngeal, ovarian, prostate, uterine, kidney, and bladder cancers (Liu H. et al., 2019; Wang et al., 2019; Wu et al., 2019; Yao et al., 2019; Yu et al., 2019; Zhang et al., 2019; Zhi et al., 2019). Moreover, it has been reported that circRNAs act as RNA sponges, binding to their corresponding miRNAs to regulate the expression of target genes (Memczak et al., 2013). Nevertheless, the aberrant expression of circRNAs on a transcriptional level as well as their regulatory functions have not been explored in PCC/PGL patients. Therefore, in this study, we conducted a comprehensive analysis of differentially expressed circRNAs and their potential epigenetic role in PCC/PGLs, subsequently constructing a circRNA-miRNA CNC network that shows the systematic regulatory function of circRNAs in the pathogenesis of PCC/PGLs.

PCC/PGLs are highly genetically related tumors, and thus research on the regulatory function of its transcription processes may further our understanding of PCC/PGL development and better contribute to the diagnosis and treatment of the disease (Neary et al., 2011; Liu et al., 2015; Zhu et al., 2019; Liu Y. et al., 2019). In the last decades, numerous studies have screened the genetic alterations of PCC/PGLs in an attempt to elucidate the major mutant genes and the disease subtypes caused by those genes (Neumann et al., 2007; Neary et al., 2011; Gill et al., 2011; Taieb and Pacak, 2017; Yi et al., 2018; Abrard et al., 2019). As PCC/PGLs are closely related to epigenetic regulation, comprehensive regulatory network studies are more conducive for explaining their pathogenesis than are studies conducted on a few molecules. Thus, we performed transcriptome analysis of seven PCC/PGL patients *via* RNA sequencing and subsequently identified 3927 mRNAs, 283 miRNAs, and 367 circRNAs that were differentially expressed. We then selected the top 11 differently expressed circRNAs for real-time PCR validation in 33 pairs of tumor tissues and adjacent normal tissues of PCC/PGL patients, and found that the cross-regulated target genes were consistent with the sequencing results.

Some studies have shown that circRNAs can regulate DNA methylation in certain diseases. Wang et al. (Morley-Smith and Lyon, 2016) reported that CircIBTK can inhibit DNA demethylation and inactivate the AKT pathway in systemic lupus erythematosus patients. Our GO and KEGG pathway analysis of the sequencing results also showed that the major differentially expressed circRNAs were enriched in methylation-related molecular functions such as histone methyltransferase activity and tRNA (cytosine-5-)-methyltransferase activity, which suggests that these circRNAs may affect the development and progression of PCC/PGLs by participating in the modification of target gene methylation. Further analysis of our miRNA sequencing data showed that the histone methylation-related miRNAs predicted by the circRNA-miRNA CNC network were indeed downregulated in tumor tissues, supporting the notion that these circRNAs and miRNAs interact with each other and regulate histone methylation. Moreover, in the KEGG pathway-enriched data, genes with higher enrichment were mainly related to insulin secretion and axon guidance, which coincides with the pathological mechanism of PCC/PGLs patients, wherein neuroendocrine tumors secrete a large amount of catecholamines to disrupt the endocrine and metabolism balance, leading to a series of clinical symptoms such as hypertension and obesity (Neary et al., 2011). Therefore, these differentially expressed circRNAs may affect the secretion and metabolism processes of PCC/PGL patients by modulating their target genes. Our findings thus provide new strategies and targets for the diagnosis and treatment of such patients.

It is well known that circRNAs are stable, specific, and abundant (Wang et al., 2018; Yu et al., 2019; Zhi et al., 2019), making them suitable for detection as circulating biomarkers in liquid biopsies. Indeed, we identified some differentially expressed circRNAs in PCC/PGL patients, such as hsa_circ_0000567, hsa_circ_0002897, and hsa_circ_0004473, that bind to miRNA and affect histone methylation; these three circRNAs were also found differentially expressed between the peripheral blood of PCC/PGL patients and healthy donors. Previous studies have shown that the hsa-miR-183/182/96 cluster, hsa-miR-21-3p, hsa-miR-551b-3p, and hsa-miR-202-5p are associated with metastatic risk and progression of PCC/PGLs (Castro-Vega et al., 2015; Calsina et al., 2019). Notably, our study showed that hsa_circ_0000567 may bind to hsa-miR-96-3p and regulate histone methylation, highlighting hsa_circ_0000567 as a diagnostic and prognostic biomarker for PCC/PGL patients. Meanwhile, *SDHx*, *MEN1*, *RET*, and *HF* susceptibility genes were identified as source genes according to our mRNA sequencing data, after which the circRNA-miRNA-mRNA CNC network of PCC/PGL patients was mapped, providing a basis as well as novel ideas for future PCC/PGL research and diagnostic biomarker discovery.

In conclusion, the present study demonstrated that differentially expressed circRNAs such as hsa_circ_0000567, hsa_circ_0002897, and hsa_circ_0004473 can interact with their target miRNAs and regulate histone methylation during the pathogenesis of PCC/PGLs. KEGG pathway analysis revealed that the differently expressed circRNAs were involved in endocrine-related pathways, consistent with the clinical manifestations of PCC/PGLs. In addition, we identified the circRNAs associated with PCC/PGL susceptibility genes and mapped their regulatory network, which may provide potential prognostic biomarkers and therapeutic targets for PCC/PGL patients.

## Data Availability Statement

The accession number for the data deposited in the GEO database is GSE139518.

## Ethics Statement

All patients signed the informed consent forms before the surgery and study, which was approved by the Ethics Committee of Xiangya Hospital of Central South University.

## Author Contributions

AY and ML designed the study and conducted the experiment and wrote down the manuscript, DC, CW, and QX collected the samples, CX help analyze the result and gave writing suggestions. LZ, YW, XZ, and YP help review the manuscript. LL guided the study. All the authors approved the final version of the manuscript.

## Funding

This work was supported by the National Natural Science Foundation of China (grant number 81400773), Innovation-Driven Project of Central South University (No.020CX046), and Funds for the Shenghua Yuying Talents Program of Central South University.

## Conflict of Interest

The authors declare that the research was conducted in the absence of any commercial or financial relationships that could be construed as a potential conflict of interest.

## Supplementary Material

Supplementary Table 1 CircRNAs on all the chromosome.

Supplementary Table 2 The information of the 33 patients.

Supplementary Table 3 The information of the 16 PCC peripheral blood.

Supplementary Table 4 PHEOCvsPHEON.differentially expressed miRNA.

Supplementary Table 5 PHEOCvsPHEON.differentially expressed Mrna.

Supplementary Table 6 Primer sequences for identification of circRNA by qRT-PCR.
