# Supplementary material for: A Comprehensive Analysis Identified the Key Differentially Expressed Circular Ribonucleic Acids and Methylation-Related Function in Pheochromocytomas and Paragangliomas
Source: Front Genet. 2020 Feb 25;11:15. doi: 10.3389/fgene.2020.00015 (PMC7052364; doi:10.3389/fgene.2020.00015)
Supplement: Supplementary Table 6 — Primer sequences for identification of circRNA by qRT-PCR. [file Table_6.docx]

**Primer sequences for identification of circRNA by qRT-PCR**

| **Primer name** | | **Sequence (5ʹ to 3ʹ)** | |
| --- | --- | --- | --- |
| hsa_circ_0000567F | | AGAATGAACAGCCCCATCTG | |
| hsa_circ_0000567R  hsa_circ_0000825F  hsa_circ_0000825R | | AGTGGGTTCCCATGAATGAA  CTCCGACAGCAGATGATTGA  CAGCCACTTTCAGGCTTTTC | |
| hsa_circ_0007444F | | CTGTTCTGCTGCCAGTGTGT | |
| hsa_circ_0007444R | | GATCCTGGGTAGTTCGGATG | |
| hsa_circ_0001573F | | CAGAAGGAACCAGGAAACGA | |
| hsa_circ_0001573R | | TCAGTGACTTTCCGCAGATG | |
| hsa_circ_0000972F | | GGAGTGGAGAACATGCACAA | |
| hsa_circ_0000972R | | GTGCAAGATAAAGGCCCAAA | |
| hsa_circ_0019773F | | AAGTTGGGAGAGGCTGGAAT | |
| hsa_circ_0019773R | | TCACGTGTCCTTTTTCACCA | |
| hsa_circ_0056892F | | AACCTCCAGCCAACACAGAG | |
| hsa_circ_0056892R  hsa_circ_0002897F | | TCAGCAAACTCTGTCGCATC  TACGTTGATGTCAAACCCCTTT | |
| hsa_circ_0002897R | | CTTGGGATGAGAGAAAGCACA | |
| hsa_circ_0003265F | | AGAATGAACAGCCCCATCTG | |
| hsa_circ_0003265R | | TGTAGTGGGTTCCCATGAATG | |
| hsa_circ_0004473F | | TTGAGGAAGGTGTAGAACACGAT | |
| hsa_circ_0004473R | | GCTTCCTTGCTGACAGACATT | |
| hsa_circ_0007279F | | CGAGATCATCACTGGTATGCC | |
| hsa_circ_0007279R | | TTGAATCTGAGAGAGGAGTATTCAGA | |
| β-actin-F | | CTCCATCCTGGCCTCGCTGT | |
| β-actin-R | | GCTGTCACCTTCACCGTTCC | |
